# Are the Chinese Moving toward a Healthy Diet? Evidence from Macro Data from 1961 to 2017

**DOI:** 10.3390/ijerph17155294

**Published:** 2020-07-23

**Authors:** Aixi Han, Tianhao Sun, Jing Ming, Li Chai, Xiawei Liao

**Affiliations:** 1 International College Beijing, China Agricultural University, Beijing 100083, China; 2017314060118@cau.edu.cn (A.H.); 1414060704@cau.edu.cn (T.S.); 2 School of Atmospheric Sciences, Chengdu University of Information Technology, Chengdu 610225, China; mingjing@cuit.edu.cn; 3 Chinese-Israeli International Center for Research and Training in Agriculture, China Agricultural University, Beijing 100083, China; 4 School of Environment and Energy, Peking University Shenzhen Graduate School, University Town, Shenzhen 518055, China; xiawei.liao@pku.edu.cn; 5 College of Environmental Sciences and Engineering, Peking University, Beijing 100871, China

**Keywords:** healthy diet, food consumption, diet balance index, dietary guidelines

## Abstract

The change in diet structure is one of the critical features of social transformation, and diet structure is directly related to human health. In China, with rapid economic development, changes in the diet structure of the population have begun and are proceeding at a fairly rapid rate. In order to reveal how the Chinese diet is approaching or deviating from the nutritional goal, a novel index, NDBI (National Dietary Balance Index), is developed in this study to investigate the Chinese diet from 1961 to 2017 at a national level. The results show that the Chinese diet has transitioned from the under-intake stage to the over-intake stage. Before the 1980s, Chinese people ate all foods inadequately except staple foods; after the 1980s, the issue of under-intake began to fade, and the intake of meats even became excessive. The intake of staple foods is always excessive during this period. Currently, the Chinese diet is still unhealthy because of the inadequate intake of dairy products and the excessive intake of staple foods and meats. By evaluating diet structure on a national level, this study can help people to better understand how the Chinese diet deviated from the nutritional goal and provides information for policymakers intervening in China’s food consumption.

## 1. Introduction

Strongly affected by regional habits and changes in social development, dietary consumption has regionality and variability, but it affects people’s quality of life and health to a large extent. A healthy and balanced diet is the key to the nutrition goal. Still, inappropriate diets may lead to the prevalence of non-communicable diseases (NCDs), such as cardiovascular disease or diabetes, which severely harm human health [1]. Based on the dietary guidelines, a scientific evaluation method can be established to measure people’s dietary quality and guide people to improve their nutritional status. The dietary quality evaluation is an essential part of the nutritional status evaluation. The most commonly used method is to use the dietary evaluation index to evaluate and calculate dietary habits and behaviors that are difficult to quantify accurately. As dietary intake plays an important role in energy balance and has a significant impact on obesity and many non-communicable diseases, it is increasingly the case worldwide that scholars often use some diet quality indexes to assess whether the diet is balanced and determine its impact on health [1,2]. In America, the US Department of Agriculture (USDA) published the Healthy Eating Index (HEI), based on the US Dietary Guidelines, to assess whether the dietary quality of US residents meets the dietary guidelines [3]. Patterson et al. established another scoring method called the Dietary Quality Index (DQI) in 1994, which was revised by Haines et al. in 1999 based on new dietary guidelines (DQI-R) [4,5]. DQI-R has ten scoring items, and there are separate tables for dietary diversity and dietary suitability. At the same time, each score value of DQI-R ranges from 0 to 10 points, with a total score of 100 points, and the higher the score value, the more the diet is in line with the dietary guidelines [5]. According to relevant research, the Mediterranean dietary pattern (MDP), popular in the Mediterranean, achieves a good balance between different foods and diet ingredients. Therefore, Panagiotakos et al. have established the Med Diet Score (MDS) for evaluating the degree to which the diet conforms to the traditional Mediterranean diet [6,7,8]. With the advancement of research on the structure of food consumption, researchers worldwide have also established a large number of other dietary quality evaluation indexes. The scoring items of these evaluation indexes are mainly divided into the food group and the nutrient group. Some indexes include both groups, such as DQI and HEI, but some indexes only consist of one group, such as DBI, which only includes food group items.

However, the design of these indexes is based on the actual diet structure and dietary habits of certain countries or regions; therefore, they are not universal. With respect to Chinese dietary habits, there are currently two diet quality indexes, the Diet Quality Index (DQI) and Diet Balance Index (DBI), both of which can effectively evaluate the dietary quality of Chinese residents. The setting of the Chinese Diet Quality Index (i.e., INFH-UNC-CH DQI) refers to the US DQI-R [9]. Its score is designed in a two-way setting, where a negative score indicates malnutrition, a positive rating indicates over-nutrition, and a zero score indicates compliance with dietary recommendations. It contains ten main scoring items, with a total score of −74–56, in which 0 is in full compliance with the dietary guidelines, and the farther the score is from 0, the lower the quality of the diet. Another index, DBI (Diet Balance Index), contains only food group items and is closely related to Chinese dietary characteristics. Compared with the use of nutrient group items to measure dietary structure, which lacks operability, the use of food group evaluation such as in the DBI can reflect insufficient dietary intake and excessive intake in a more acceptable way [10]. 

With rapid economic and social development, food consumption in China has undergone tremendous changes. The consumption pattern that used to be grain-based is gradually transitioning to a consumption pattern that is based on high-value foods such as meat, dairy products, and aquatic products, and, at the same time, residents are beginning to pay attention to the topic of healthy dietary structure [11,12,13]. Chen concludes that the main and ultimate concern of Chinese citizens is a high-quality diet—that is, a quality-oriented pattern [14,15]. In this process, it is shifting towards diversification and nutrition, and, at the same time, residents are beginning to pursue food safety and health. From 1984 to 2017, the demand from Chinese residents for grains is decreasing, while the demand for processed foods and supplementary foods is increasing, and it is expected that the consumption of meat and dairy products will continue to rise for a long time [16,17]. Cao et al. conclude that from 1961 to 2017, the per capita energy intake of Chinese residents has more than tripled. At the same time, food consumption has shifted from cereals to meat products, especially red meats [18]. Therefore, at present, China’s food consumption problem has gradually changed from an absolute quantity shortage to a structural imbalance, and one of the most important issues is the excessive intake of meat. High levels of meat, especially red meat or processed meat consumption, have a strong positive correlation with cancer incidence [19,20,21]. At the same time, relevant research shows that the prevalence of overweight continues to rise and has become a serious threat to personal health and a major public health challenge in China [22]. Therefore, it is imperative to vigorously advocate for and promote healthy diets and adjust the dietary structure of our residents. 

Referring to the dietary guidelines of other countries, we can find that, with global nutritional changes, people’s attention to dietary problems has gradually shifted from malnutrition to overall diet quality and nutrition-related chronic diseases caused by nutritional imbalances [23,24,25]. In fact, in China, the Chinese Nutrition Society has also issued the Chinese Dietary Guidelines to popularize nutrition knowledge, guiding people to choose a healthy dietary structure and achieve a balanced diet [26]. In the latest version (2016 version), the guidelines call on everyone to change their diets and eat more fruits, vegetables, and egg-milk, with fewer meats or cereals, after considering the overall problem [27,28]. At the same time, by quantifying the overall food consumption level in China, we can grasp the dietary changes of residents so as to better provide support for improving the diet structure. Through research, Chai et al. found that the food consumption of residents is significantly affected by income, and they also emphasize the regional characteristics of such consumption: there are obvious differences between provinces and cities [29]. At present, many studies are based on the analysis of specific groups or regions. For example, Zang et al. used DBI scores to evaluate dietary quality across seasons in Shanghai, China [30]; Wang et al. evaluated Chinese dietary habits by focusing on the obesity rate [31]; Meng et al. discussed the nutritional problem among Chinese children caused by micronutrient inadequacy [32]. However, it is also very important to understand whether the overall dietary structure of Chinese people can guarantee human health from a macro perspective, which is an important basis for policymakers to refer to. In fact, at present, a macro indicator that can measure the overall national dietary structure is lacking; inquiring into the overall food consumption of the Chinese people from a macro perspective can allow us to better grasp the general trend of food consumption for the entire population and explore the main problems in the transformation of the food consumption structure, thus bringing scientific suggestions for the improvement of residents’ food consumption. Therefore, this paper proposes a DBI scoring system and focuses on the national level to study whether the food consumption in China has become healthier and more balanced. The purpose of this study is to use quantitative evaluation methods to test the diet balance of Chinese residents and use macro data to reveal the dietary health of Chinese residents over the past 52 years, so as to provide theoretical support for improving the dietary structure of residents.

## 2. Materials and Methods 

**Figure 1:**
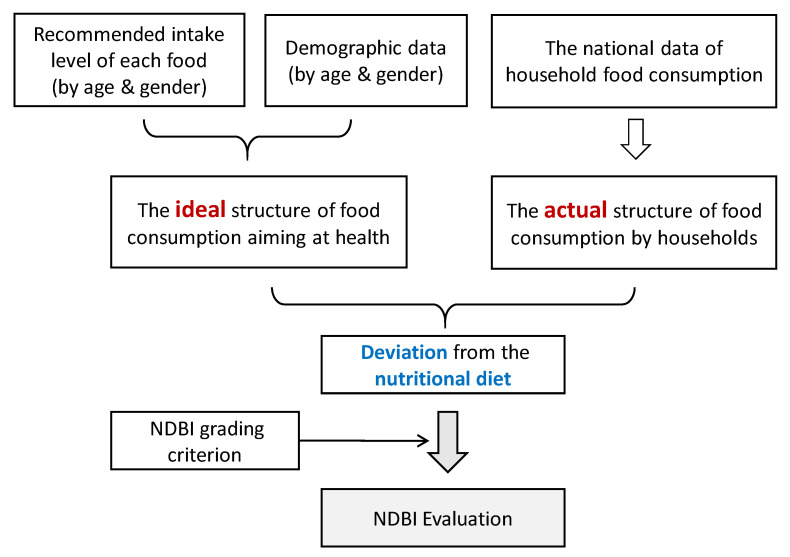
Structural flow diagram of evaluating National Diet Balance Index (NDBI).

As shown in Figure 1, the National Diet Balance Index (NDBI) is evaluated based on the deviation of the actual food consumption from a nutritional diet. Nutritional diet indicates the food consumption of healthy eating and can be calculated according to the recommended diet for each age–gender group and the demographic data. The period of1961 to 2017 is chosen to be studied according to the data available in the FAO (Food and Agriculture Organization of the United Nations) database.

### 2.1. NDBI Grading System

The food categories of NDBI are set based on the Balanced Dietary Patterns from the Chinese Dietary Guidelines, which are divided by different food groups—that is, different calorie required levels [26]. Chinese Dietary Guidelines also provide us with the EER (estimated energy requirement), which measures the calorie intake that meets minimum health standards [26], therefore providing us with the recommended calorie levels for different ages and genders. Combining the two, we can obtain a combination of foods recommended for different ages and genders. 

In the Chinese Dietary Guidelines, there are four main food categories, including C1 (cereals, legumes, and tubers), C2 (vegetables and fruits), C3 (red meats, poultry, eggs, and aquatic products), and C4 (dairy products, soybean, and nuts). All these foods are listed in units of grams per day. 

The basis of constructing the NDBI index is similar to that of producing DBIs. For a DBI, each individual with different suggested intake levels can have their own corresponding recommended food intake level in each category. On the other hand, an NDBI index is a macroeconomic study, so the recommended food intake level by category, in each year, should have its own corresponding amount, which can represent that year’s recommended intake level as a whole.

In order to obtain a national dietary evaluation score, we need to set a dietary recommendation standard, which is based on the national population’s age–gender structure and dietary combinations corresponding to different age–gender groups. At the same time, World Population Prospects provides detailed population data, so we can obtain the population structure of China from 1961 to 2017 (by gender and age) [33]. Therefore, by combining these two functions, we can find the overall recommended intake levels for Chinese residents in general, including all age groups and genders. The equation is as follows:

(1) $$I_{rec,i}=\frac{\sum_{j}\left(I_{j}\times P_{ij}\right)}{P_{i}}$$

where, $I_{rec,i}$ indicates the national recommended intake level in year *i*;$I_{j}$ indicates the intake level recommended by the Chinese dietary guidelines in *j* age–gender group; $P_{ij}$ indicates the number of populations in the j age–gender group in year i; $P_{i}$ indicates the total number of populations in year i.

### 2.2. NDBI Scores

The NDBI scores in year *i* can be calculated using the following equation:

(2) $$NDBI_{i}=\{\begin{array}{c} UP,\frac{I_{act,i}-I_{rec,i}}{I_{rec,i}}\geq 1\\ UP\times \frac{I_{act,i}-I_{rec,i}}{I_{rec,i}},\;0\leq \frac{I_{act,i}-I_{rec,i}}{I_{rec,i}}\leq 1\\ LO\times \frac{-I_{act,i}+I_{rec,i}}{I_{rec,i}},\;-1\leq \frac{I_{act,i}-I_{rec,i}}{I_{rec,i}}\leq 0\\ LO,\;\frac{I_{act,i}-I_{rec,i}}{I_{rec,i}}\leq -1 \end{array}$$

where *UP* and *LO* are respectively the upper limit and the lower limit of the score range for each food item, as shown in Table 1; $I_{rec,i}$ is calculated in Equation (1), which indicates the national recommended intake level in year i; $I_{act,i}$ represents the actual average intake on the national level in year i—that is, the food consumption data obtained from the FAO database [34]. 

### 2.3. NDBI Evaluation

Similar toDBI_16, there are four sub-indexes in the NDBI system—namely total score (TS), which reflects the overall diet balance, high bound score (HBS), which investigates the excessive intake, low bound score (LBS), which examines the inadequate intake, and diet quality distance (DQD), which reveals the degree of deviation from nutritional diet [10].

- TS (total score): adding up both negative scores and positive scores to reflect the overall diet balance. A negative TS indicates an overall under-intake diet; a positive TS indicates an overall over-intake, unbalanced diet. If the score is 0, it does not necessarily indicate that the overall intake is balanced, since it could be that the over-intake effect balanced out the under-intake effect.
- DQD (diet quality distance): adding up the absolute values of both negative scores and positive scores to reflect the degree of diet imbalance for the whole diet—the higher the score, the higher the degree of intake imbalance.
- HBS (high bound score): adding up all the positive scores to reflect the degree of over-intake in the whole diet.
- LBS (low bound score): adding up all the absolute values of negative scores to reflect the degree of under-intake in the whole diet.

## 3. Results

Through calculations, we finally obtain the NDBI scores of different food types from 1961 to 2017, which are used to observe the trend of the food consumption balance of various food categories of Chinese households in the past 57 years. 

### 3.1. Staple Foods

In this category, there are three kinds of foods: cereals, pulses, and tubers. The score of this category did not decline until 2006, but this is only by less than one point, which shows the problem of the over-intake of staple foods. From the data of the FAO, we can see that the consumption of cereals is increasing year by year and the consumption of pulses is decreasing year by year, but they both exceed the recommended levels of dietary guidelines. At the same time, the consumption of tubers has been less than the recommended amount in a diet. As a result, due to the overconsumption of cereals and pulses, whose influence on the NDBI score of staple foods is far greater than that of tubers, the NDBI score of staple foods is never lower than 12. This poor situation has continued: even after 2006, the NDBI score of staple foods has declined, but it still presents the serious problem of excessive intake. Therefore, we can reach the conclusion that the over-intake of cereal and tubers remains a common problem, while pulse consumption is faced with the problem of insufficient intake. This result is similar to the conclusion made by Du et al. and Huang et al. [35,36]. All of these lead to the excessive intake of staple foods, which needs special attention in the dietary structure of Chinese residents.

### 3.2. Fruits and Vegetables 

**Figure 2:**
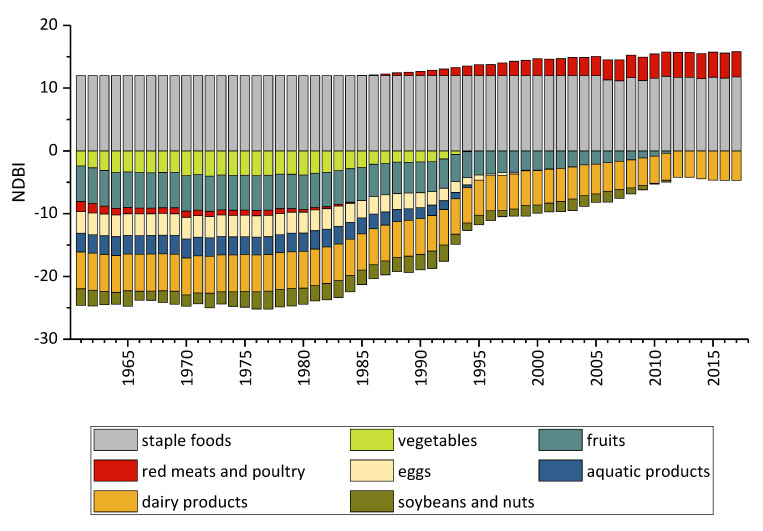
NDBI scores for each food group.

According to Figure 2, we can see that the consumption of vegetables and fruits has increased year by year after experiencing negative scores. Among them, the vegetable NDBI score began to increase rapidly after a brief decrease in 1980, reaching the highest score in 1996(0 point). The score of fruit has always been on the rise, and its growth rate has accelerated significantly since 1980, showing a “J” type development, reaching the highest score of 0 in 2012.

It can be seen that the NDBI score change trends of vegetables and fruits are the two best-performing values in the classification. In the 1960s, there are major problems with food consumption in both categories: the degree of deviation for vegetables is about 40% while the degree of deviation for fruits is about 94%, indicating a serious problem of under-intake. However, with the rapid development of China’s agricultural technology and food consumption system, both have steadily risen to a balanced intake level—that is, they have reached 0 points. Vegetable intake reached the equilibrium threshold in 1995, and fruit intake reached the equilibrium threshold in 2012. This shows that the intake of vegetables and fruits of Chinese nationals has gradually increased from the initial lack, and a basic balance has been achieved.

### 3.3. Animal Products

The category of animal products includes red meats and poultry, eggs, aquatic products, and dairy products. It can be seen from Figure 2 that all four curves show an upward trend. The score for red meats and poultry has risen from −1 in 1961 to 0 in 1985 and kept this tendency to 4 in 2017. The score for eggs increased from −3.5 in 1961 to 0 in 2003 and has been maintained ever since. At the same time, its growth rate is slow at first and then became faster, accelerating in the 1980s. The growth trend of aquatic products is roughly the same as that of eggs, rising from −3 in 1961 to 0 in 1995 and remaining stable since then, also accelerating in the 1980s. The growth rate of dairy products is different from the previous three, and its score increased slowly. In 1961, the score is−6, and by the beginning of the 21st century (i.e., about the 2000s), it had only increased to about −5.5. Although acceleration happened during 2000 and 2006, and the score reached 5 in2017, the score is still negative.

It can be seen from Figure 2 that animal products have experienced serious problems, transitioning from under- to over-intake in the past 50 years. In the 1970s, the consumption of red meats and poultry experienced a stable development stage. After the 1980s, the overall demand for red meats and poultry grew rapidly, which is closely related to the overall social development of China. At the same time, it should be noted that, in 1985, red meats and poultry reached a balanced level, consistent with the recommended intake for active livestock and poultry consumption. However, this result did not last long. By the beginning of the 21^st^ century, the consumption of livestock and poultry is more than twice the recommended level, and by the 1920s, this score reached 4. This reflects the problem of excessive intake of livestock and poultry among Chinese residents.

As for eggs, in 1961, we can see that the intake of eggs is more than 80% lower than the recommended level, and this lack of intake began to ease in 1985. The egg consumption trend is similar to the trend of aquatic products, and aquatic products reached a balanced intake level in the early 21st century. Through data calculations, it can be found that, since 2003, egg intake has experienced a problem, but this problem is not serious, because the actual intake is only 18% higher than the recommended intake, which is within the acceptable range, so it can be treated as a slight overdose problem. 

For dairy products, we can see from Figure 2 that the consumption of dairy products in China has been below the recommended level and its growth has been extremely slow. Even in the 2010s, the consumption of dairy products is still lower than 70% of the recommended intake. Overall, the score for dairy products in the past 50 years has only increased by 1.6 points, so the consumption of dairy products has a problem of insufficient intake. This problem can be explained by lactase intolerance, which is considered one of the most important reasons for the low consumption of milk in China [37] because the prevalence of lactase deficiency among Asians is 76% to 100%, which is much higher than that among Caucasians (5–30%), as research has concluded [38]. Therefore, providing other types of dairy products instead of milk or changing the supplied style may increase dairy consumption in a more reasonable way.

### 3.4. Soybean and Nuts

As can be seen from Figure 2, from 1961 to 2017, the NDBI score of soybean and nuts are negative, and there are some fluctuations. The score of soybean and nuts is around −3 in the 1970s–1990s, but it then rose slowly, tending towards 0, and remained at 0 points until 2012. It can be seen that the problem of insufficient intake of soybean and nuts that occurred before the 1990s is gradually alleviated afterward. After 2012, the problem of the insufficient intake of soybean and nuts is resolved, and the food consumption of soybean and nuts meets the requirements of the Chinese Dietary Guidelines.

### 3.5. Overall Evaluation

**Figure 3:**
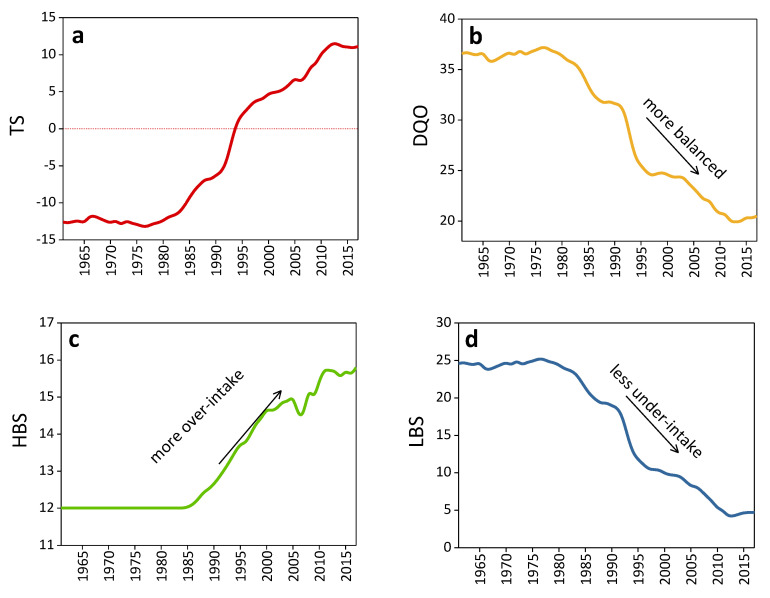
Evaluation the Chinese diet from 1961 to 2017 using NDBI system. (**a**)Total score; (**b**) diet quality distance; (**c**) high bound score; (**d**) low bound score.

The NDBI overall grade score set including LBS, HBS, TS, and DQD shows the overall evaluation of the Chinese resident’s diet balance. Figure 3 below illustrates the trend line of each score band.

#### 3.5.1. TS (Total Score)

The TS (total score) curve reflects the average level of overall dietary quality. Overall, the TS curve has been on the rise. In the past 50 years, TS remained relatively stable from 1961 to 1983, with an average value of 13, and then maintained a quick upward trend. After an extremely rapid increase from 1988, it reached 0 in 1994, which means an overall balance. In 2013, it reached its maximum, which isaround12, followed by a slow decline afterward. Finally, in 2017, TS reached 11. Therefore, from 1961 to 2017, the food consumption of Chinese residents has undergone a significant transition from insufficiency to surplus, showing the process of the imbalance of food consumption structure from improvement to deterioration. 

Overall, the first 20 years of stabilization were due to the checks and balances caused by the excessively high staple food scores. At that time, the food consumption of Chinese residents was dominated by staple foods, and the other food supplies were not enough to reach the recommended intake level provided by the dietary guidelines. After this, although the score of the staple food remained unchanged, the consumption of other foods steadily increased, especially when the score of red meat and poultry changed from negative to positive, and the score of fruit tended towards 0, etc., which promoted the rapid growth of TS and reached 0. Since then, the food consumption pattern of Chinese residents has changed from a level of inadequate intake to a level approaching food overconsumption.

#### 3.5.2. DQD (Diet Quality Distance)

DQD adds the absolute value of each index score to reflect the degree of imbalance of dietary intake. The higher the score is, the more imbalanced the diet is. Overall, from 1961 to 2017, DQD scores showed a downward trend, but the scores remained above 20, indicating that the country’s dietary imbalance improved, but there are still serious problems.

As can be seen from Figure 3, from 1961 to 1977, the DQD curve showed a relatively stable and constant trend; however, this trend ended before the 1980s. Since then, the DQD score gradually declined until it reached 20 in 2017. This change is due to the improved food consumption in all negative score zones, which lowers the DQD. It should also be noted that the reason for the improvement in the DQD score also changes with time. On the one hand, the improvement in the consumption of fruits, vegetables, aquatic products, soybean and nuts, and eggs is obvious. On the other hand, the absolute value of the score of red meat and poultry experienced a shift from falling to rising, which caused the downward trend of the DQD curve to slow down after the 2000s. In general, from 1961 to 2017, DQD dropped from 37 to 20, reflecting the improvement in the country’s household food consumption, but its consumption pattern is still in a state of imbalance.

#### 3.5.3. HBS (High Bound Score)

The HBS curve adds up the positive scores of all indicators to reflect the degree of excessive intake in the diet. From 1961 to 1984, HBS is in a stable state, maintaining a score of 12. After 1985, the situation changed and HBS gradually increased, reaching 16 in 2017. Although there is a slight decline around 2004, the HBS curve is generally J-shaped, showing a trend from slow to fast growth. It is shown that the excessive intake of staple foods led to the formation of HBS, and after the 21st century, the problem of the excessive intake of livestock and poultry became more and more serious, resulting in an increase in around3% points in HBS. Therefore, overall, the problem of excessive intake in the diet becomes more serious as the intake of red meats and poultry increases.

#### 3.5.4. LBS (Low Bound Score)

The LBS curve is formed from the absolute value of the negative scores in all indicators, thus reflecting the problem of insufficient intake of diet. In general, LBS experienced a process of keeping constant first and then falling. LBS maintained a score of around 24 from 1961 to 1980, and, after that, it faced a decline, where, finally, in 2017, the HBS is around 4.7. When looking at the specific scores, we can see that the scores for other kinds of foods except staple foods are basically below zero, and the consumption of dairy products has contributed largely to LBS. Therefore, compared with 1961, the LBS in 2017 decreased by about 19 points, showing that the under-take problem is alleviated.

## 4. Discussion 

### 4.1. Strengths of Developing a New Dietary Evaluation System

Firstly, this article’s innovation is to establish a new macro-level indicator(NDBI) to examine the nutritional quality of the Chinese diet. Among the dietary evaluation methods, the most commonly used method is the index method, and the traditional dietary evaluation method is based on a single nutrient index, which can only reflect the status and problems of certain aspects of the diet and is not enough to explain the complex interactions between various nutrients. In order to overcome the limitations, many researchers have approaches this from different angles and established a variety of composite indicators in their diet evaluation index. For example, Gerber MJ has established MDQI (Mediterranean Diet Quality Index) to evaluate the degree to which the diet conforms to the traditional Mediterranean diet. However, its establishment is based on the dietary pattern of a specific region and does not represent the local Chinese dietary culture, so it is not universal and not suitable for Chinese dietary research. Patterson and others in the United States established the DQI (Diet Quality Index) and derived a version of the index [4]. However, because the scoring system includes nutrient intake, such data is more difficult to calculate and organize, so it may lead to deviations in the results. Therefore, the establishment of NDBI to evaluate and analyze the dietary structure at the national level is significant.

Moreover, the NDBI scoring system is based on the relevant data of DBI_16, so it has certain scientific practicality. DBI_16 is an improvement on the previous version of DBI. According to the core items in the dietary guidelines, we select the individual food indicators that constitute the dietary balance index and use the diet quality distance methods for scoring and evaluation. The scoring method for each indicator is determined with reference to the food intake recommended by the balanced diet pagoda. The definition and evaluation method of DBI_16 can better reflect the basic principles of the new Chinese dietary guidelines, which can reflect both insufficient intake and excessive intake and can be used to evaluate the dietary nutrition status of groups and individuals. It is also noticed that DBI only uses the food group as a scoring item, without complicated calculation of nutrients. Its indicators can reflect both inadequate intake and excessive intake. At the same time, because the scores of DBI indicators are set according to the nutrition tower, and the recommended intake of the nutrition tower is based on the needs of normal adults, DBI is only applicable to the dietary evaluation of adults, which also meets the requirements of this study. Therefore, the NDBI scoring system based on DBI_16 is equally scientific.

### 4.2. The Implication of the Findings for the Health Profile of Chinese Population

While NDBI quantitatively analyzes China’s dietary consumption structure, it also reveals the changes in dietary structure over time and assesses such changes. As the most populous country in the world, China has undergone major changes in its economic development and diet structure in the past few decades. Simultaneously, as in other developing countries, the modernization of the dietary structure has had a profound impact on people’s health [1]. With the transformation of the diet structure, a country that once faced widespread food insecurity and widespread malnutrition must now cope with obesity and other non-communicable diseases related to diet (e.g., type-2 diabetes, stroke, or coronary heart disease), increasing the health burden [39,40,41,42]. While non-communicable diseases are spreading, some people still lack micronutrients such as iron, vitamin A, or zinc [43]. Therefore, China is also facing the dual challenge of malnutrition and over-nutrition and urgently needs to take effective countermeasures to deal with the socio-economic burden caused by these food security challenges.

The same problem arose in Latin America. According to research by Popkin et al., in the 1980s, the eating habits of the region began to change, and this change accelerated in the following decades [44]. It is manifested by the rapid increase of fast food and the decline of traditional coarse grain consumption. Overall, dietary changes and reduced physical activity have led to a high level of adult obesity in Latin America and a rapid increase in childhood obesity rates. Complications of obesity, including diabetes and other health problems, are emerging in children and adolescents. By analyzing the forming factors of the problem from the perspective of dietary structure, in their conclusion, Popkin et al. claimed that the transformation of the food system and dietary structure is the primary condition for improving people’s health in Latin America [44].

This also has some implications for the problems in China’s existing dietary consumption structure. Many diet-related diseases are due to changes in China’s diet. The consumption of vegetable oils, animal-derived foods (pork, eggs, dairy products, etc.), and processed foods rich in refined starch, sugar, salt, and unhealthy fats continued to increase [35,45]. The consumption of staple foods gradually changed from traditional coarse-grain staple foods (e.g., sorghum) to refined cereal foods (e.g., polished rice), which also led to the problem of micronutrient deficiency [43]. Therefore, when planning the development strategy of the agricultural industry, the government needs to go beyond merely ensuring the self-sufficiency of national grain production. They should take nutrition into consideration when devising the national food security strategy to promote the improvement of dietary diversity and the improvement of the food consumption structure.

## 5. Conclusions

It can be seen from the study that, although the NDBI scores of different food groups represent improved or deteriorated tendency, in general, the Chinese diet transition is deviating from the nutritional goal. At present, China’s food shortage problem has gradually changed from an absolute quantity shortage to a structural shortage. The structural imbalances in the dietary consumption in China have seriously threatened the national health. Compared with the 1990s, China’s dietary structure has undergone a major change, from mainly tuber crops to more meat, and the food consumption problem has shifted from the lack of absolute calories in the early days of the founding of the PRC (People’s Republic of China) to the imbalance of nutritional structure is mainly manifested by the coexistence of over-nutrition and malnutrition, in which the consumption of red meats is seriously excessive. Therefore, it is imperative to vigorously advocate and promote healthy diets and adjust the diet structure of our residents. Considering that changes in the residents’ food consumption structure significantly affect residents’ health, the country should regard food consumption and nutritional health as an important factor when formulating agricultural development strategies. In the future, when China’s food system is able to provide a sufficient amount of food, the results of the food consumption assessment in this study can provide guidance for Chinese officials to guide citizens to choose the correct food that can improve the balance of their dietary intake.

## Figures and Tables

**Table 1:** The score range for each food item in the NDBI scoring system. Each food is recommended at moderate level or encouraged level. Moderate level considers both inadequate and excessive intake, so could be positive or negative, while encouraged level only considers inadequate intake. The scores are set according to DBI_16 (Diet Balance Index, 2016 version).

Food Groups	Food Items	Score Range	Recommended Level
C1	cereals	(−12) to 12	moderate
	legumes (excl. soybean)		
	tubers		
C2	vegetables	(−6) to 0	encouraged
	fruits	(−6) to 0	encouraged
C3	red meats and poultry	(−4) to 4	moderate
	eggs	(−4) to 4	moderate
	aquatic products	(−4) to 0	encouraged
C4	dairy products	(−6) to 0	encouraged
	soybean and nuts	(−6) to 0	encouraged

## References

[1] Schwingshackl L., Bogensberger B., Hoffmann G. (2018). Diet Quality as Assessed by the Healthy Eating Index, Alternate Healthy Eating Index, Dietary Approaches to Stop Hypertension Score, and Health Outcomes: An Updated Systematic Review and Meta-Analysis of Cohort Studies. J. Acad. Nutr. Diet..

[2] Reicks M., Trofholz A.C., Stang J.S., Laska M.N. (2014). Impact of cooking and home food preparation interventions among adults: Outcomes and implications for future programs. J. Nutr. Educ. Behav..

[3] Kennedy E.T., Ohls J., Carlson S., Fleming K. (1995). The healthy eating index: Design and applications. J. Am. Diet. Assoc..

[4] Patterson R.E., Haines P.S., Popkin B.M. (1994). Diet quality index: Capturing a multidimensional behavior. Am. Diet. Assoc..

[5] Haines P.S., Siega-Riz A.M., Popkin B.M. (1999). The diet quality index revised: A measurement instrument for populations. J. Am. Diet. Assoc..

[6] Richter C.K., Skulas-Ray A.C., Kris-Etherton P.M. (2014). Recent Findings of Studies on the Mediterranean diet: What are the implications for current dietary recommendations?. Endocrinol. Metab. Clin..

[7] Mattioli A.V., Palmiero P., Manfrini O., Puddu P.E., Nodari S., Dei Cas A., Mercuro G., Scrutinio D., Palermo P., Sciomer S. (2017). Mediterranean diet impact on cardiovascular diseases: A narrative review. J. Cardiovasc. Med..

[8] Panagiotakos D.B., Pitsavos C., Stefanadis C. (2006). Dietary patterns: A Mediterranean diet score and its relation to clinical and biological markers of cardiovascular disease risk. Nutr. Metab. Cardiovasc. Dis..

[9] Stookey J.D., Wang Y., Ge K., Lin H., Popkin B.M. (2000). Measuring diet quality in China: The IN-FH-UNC-CH diet quality index. Eur. J. Clin. Nutr..

[10] Xuan D., Gengsheng H. (2009). A comparative study of healthy diet index and Chinese diet balance index. Health Res..

[11] Zhihao Z., Ying G., Yinyu Z. (2016). The impact of income growth on the food consumption patterns of urban residents. Economics.

[12] Zhang W., Shen G., Cao H., Xu X., Wang H. (2016). Major Agricultural Products Consumption Trend, Influence and Policy: During the 13^th^ Five-year Period. Agric. Econ. Issues..

[13] Huang J. (2018). Forty Years of China’s Agricultural Development and Reform and the Way Forward in the Future. Agric. Technol. Econ..

[14] Chen Z.M., Guo Q.H., Jiang H.M. (2018). Resident Food Consumption Upgrade and Chinese Agricultural Transformation. Mod. Econ. Res..

[15] Zhang Y., Tian Q., Hu H., Yu M. (2019). Water Footprint of Food Consumption by Chinese Residents. Int. J. Environ. Res. Public Health.

[16] Lv X., Li L., Liu M.L., Wei S.W., Wang S.M., Fan D.Q. (2017). Comparative Analysis of Food Consumption Transformation Characteristics of Chinese Urban and Rural Residents from 1984 to 2014. J. Shandong Agric. Univ..

[17] Xin L., Li P. (2018). Food Consumption Patterns of Chinese Urban and Rural Residents Based on CHNS and Comparison with the Data of National Bureau of Statistics. J. Nat. Resour..

[18] Cao Y., Chai L., Yan X., Liang Y. (2020). Drivers of the Growing Water, Carbon and Ecological Footprints of the Chinese Diet from 1961 to 2017. Int. J. Environ. Res. Public Health.

[19] Crippa A., Larsson S.C., Discacciati A., Wolk A., Orsini N. (2018). Red and Processed Meat Consumption and Risk of Bladder Cancer: A dose–response Meta-Analysis of Epidemiological Studies. Eur. J. Nutr..

[20] Farvid M.S., Stern M.C., Norat T., Sasazuki S., Vineis P., Weijenberg M.P., Wolk A., Wu K., Stewart B.W., Cho E. (2018). Consumption of Red and Processed Meat and Breast Cancer Incidence: A Systematic Review and meta-analysis of Prospective Studies. Int. J. Cancer.

[21] Squires J., Roebothan B., Buehler S., Sun Z., Cotterchio M., Younghusband B., Dicks E., Mclaughlin J.R., Parfrey P.S., Wang P.P. (2010). Pickled Meat Consumption and Colorectal Cancer (CRC): A Case-Control Study in New found land and Labrador, Canada. Cancer Causes Control.

[22] Ren Y., Li H., Wang X. (2019). Family Income and Nutrition-Related Health: Evidence from Food Consumption in China. Soc. Sci. Med..

[23] Ponce X., Rodriguez-Ramirez S., Mundo-Rosas V., Shamah T., Barquera S., Gonzalez de Cossio T. (2014). Dietary quality indices vary with sociodemographic variables and anthropometric status among Mexican adults: A cross-sectional study. Results from the 2006 National Health and Nutrition Survey. Public Health Nutr..

[24] Van der Horst K., Brunner T.A., Siegrist M. (2011). Fast food and take-away food consumption are associated with different lifestyle characteristics. J. Hum. Nutr. Diet..

[25] Chang J., Wang Y. (2016). Chinese Nutrition and Health Surveillance 2010–2013.

[26] (2016). Chinese Nutrition Society. https://www.cnsoc.org.

[27] Van Mierlo K., Rohmer S., Gerdessen J.C. (2017). A model for composing meat replacers: Reducing the environmental impact of our food consumption pattern while retaining its nutritional value. J. Clean. Prod..

[28] Li Y., Wang D.D., Ley S.H., Howard A.G., He Y., Lu Y., Danaei G., Hu F.B. (2016). Potential Impact of Time Trend of Life-Style Factors on Cardiovascular Disease Burden in China. J. Am. Coll. Cardiol..

[29] Chai L., Han Z., Liang Y., Su Y., Huang G. (2020). Understanding the Blue Water Footprint of Households in China from a Perspective of Consumption Expenditure. J. Clean. Prod..

[30] Zang J., Yu H., Zhu Z., Lu Y., Liu C., Yao C., Bai P., Guo C., Jia X., Zou S. (2017). Does the dietary pattern of shanghai residents change across seasons and area of residence: Assessing dietary quality using the Chinese diet balance index (DBI). Nutrients.

[31] Wang J., Yan S., Xiao H., Zhou H., Liu S., Zeng Y., Liu B., Li R., Yuan Z., Wu J. (2017). Anti-Obesity Effect of a Traditional Chinese Dietary Habit-Blending Lard with Vegetable Oil while Cooking. Sci. Rep..

[32] Meng L., Wang Y., Li T., Loo-Bouwman C.A.V., Zhang Y., Man-Yau Szeto I. (2018). Dietary Diversity and Food Variety in Chinese Children Aged 3–17 Years: Are they Negatively Associated with Dietary Micronutrient Inadequacy?. Nutrients.

[33] United Nations, Department of Economic and Social Affairs, Population Division (2019). World Population Prospects 2019.

[34] FAO Actual Chinese Household Consumption Data from 1961 to 2017. Food and Agriculture Organization of United Nations (FAO), 1961–2017, Rome.

[35] Du S.F., Wang H.J., Zhang B., Zhai F.Y., Popkin B.M. (2014). China in the period of transition from scarcity and extensive undernutrition to emerging nutrition-related non-communicable diseases, 1949–1992. Obes. Rev..

[36] Huang C., Lu Y., Zang J., Wang Z., Zhou J., Zhu Z., Zou S. (2016). The Trend in Dietary Structure and Nutrition Transition among Residents in Shanghai, from 1982 to 2012. J. Environ. Occup. Med..

[37] Wang Y., Li S. (2008). Worldwide trends in dairy production and consumption and calcium intake: Is promoting consumption of dairy products a sustainable solution for inadequate calcium intake?. Food Nutr. Bull..

[38] Vandenplas Y. (2015). Lactose intolerance. Asia Pac. J. Clin. Nutr..

[39] Popkin B.M. (2014). Synthesis and Implications: China’s Nutrition Transition in the Context of Changes Across Other Low- and Middle-Income Countries. Obes. Rev. Off. J. Int. Assoc. Study Obes..

[40] Popkin B.M., Du S. (2003). Dynamics of the nutrition transition toward the animal foods sector in China and its implications: A worried perspective. J. Nutr..

[41] Zhai F.Y. (2014). Dynamics of the Chinese diet and the role of urbanicity, 1991–2011. Obes. Rev..

[42] Popkin B.M., Keyou G., Zhai F., Guo X., Ma H., Zohoori N. (1993). The nutrition transition in China: A cross-sectional analysis. Eur. J. Clin. Nutr..

[43] Chang X., DeFries R.S., Liu L., Davis K. (2018). Understanding Dietary and Staple Food Transitions in China from Multiple Scales. PLoS ONE.

[44] Popkin B.M., Reardon T. (2018). Obesity and the Food System Transformation in Latin America: Obesity and Food System Transformation. Obes. Rev..

[45] Du S., Mroz T.A., Zhai F., Popkin B.M. (2004). Rapid income growth adversely affects diet quality in China-particularly for the poor. Soc. Sci. Med..

